# A Web-Based Gaming Approach to Decrease HIV-Related Stigma: Game Development and Mixed Methods Evaluation

**DOI:** 10.2196/37219

**Published:** 2022-12-15

**Authors:** Xiaoxiao Zhang, Erman Lai

**Affiliations:** 1 School of Journalism and Communication Jinan University Guangzhou China

**Keywords:** serious games, interactive narrative games, games for health, entertainment education, digital health intervention, game development and evaluation, mixed methods evaluation, participatory culture, people living with HIV, HIV-related stigma, intimacy stigma

## Abstract

**Background:**

The stigma faced by people living with HIV causes difficulties in the treatment of HIV/AIDS. Decreasing this stigma is thus no less urgent than implementing behavioral interventions. Serious games are being increasingly adopted as an intervention mechanism to control HIV/AIDS around the world. However, the development and evaluation of these games in China are far from adequate.

**Objective:**

This research aimed to help decrease HIV-related stigma in China via the development and evaluation of a serious game, as well as promote a participatory gamification culture for health interventions.

**Methods:**

Initially, a serious game was developed using free resources from a user-generated content website. Then, quantitative and qualitative methods were employed for game evaluation. A randomized controlled trial was conducted to explore the game’s effect on HIV-related stigma. The trial included 167 university students, who were randomly allocated to game and control groups. After the experimental evaluation, focus group discussions were held with 64 participants, who were invited to form 16 groups.

**Results:**

The game was called The Second Kind of Life with HIV (SKLWH), which is a free online game that can be played on computers and smartphones. This game hopes to publicize that people living with HIV can live a normal life, that is, a second life different from that imagined by the public. Based on the gamification practice of SKLWH, the participatory serious game development model (PSGDM) was proposed, which guided the development of 3 other HIV-themed games. The trial showed that intimacy stigma was much more severe than morality stigma and personal interaction stigma. Females were more tolerant of morality stigma than males (mean score: 1.29 vs 1.50; *P*=.01). The game intervention showed an advantage in decreasing intimacy stigma (mean score [game vs control]: 2.43 vs 2.73; *P*=.04). The group discussions validated the quantitative results and provided further in-depth information. The game intervention was largely preferred by participants, and the belief in intimacy impossibility was commonly expressed by participants when considering their relationship with people living with HIV.

**Conclusions:**

HIV/AIDS education should adopt appropriate media interventions to mitigate different dimensions of HIV-related stigma. Serious games should be used to decrease intimacy stigma, which is the hardest form to diminish. It is expected that the PSGDM can promote the development of more health games. Furthermore, HIV/AIDS intervention requires interdisciplinary efforts and cooperation that will allow more people to participate and share the responsibility of promoting health.

## Introduction

### Background

Despite progress in HIV/AIDS prevention and control, its prevalence in China has not declined [1]. In 2020, the death toll of AIDS in the country was 4 times that of COVID-19 [2]. In the past 3 years, it has always ranked first among class-A and class-B infectious diseases [2,3]. People living with HIV in China are, to a large extent, ostracized by the public as “others.” This stigma causes difficulties in the prevention and treatment of the disease. Decreasing this stigma is thus no less urgent than implementing behavioral interventions.

In HIV/AIDS education for young people, digital games are an intervention strategy with great potential [4]. These games are increasingly adopted in the international academic context [5-8]. In China, HIV/AIDS educational games are in their infancy. Besides the games developed by the team of the author XZ, there is only a short list of serious games that focus on HIV/AIDS, including Winter AIDS: a Wondrous Journey, Crossroad in Life Path, Waterloo Bridge Café, and AIDS Fighter · Health Defense. Furthermore, HIV/AIDS-themed games, both in China and abroad, usually focus on knowledge education, behavioral change, and antiretroviral treatment uptake. Very few games focus on decreasing HIV-related stigma. As an interdisciplinary attempt, this research is among the first in China to use a web-based gaming approach to mitigate stigma and help diminish the prevalence of HIV/AIDS.

In this research, a serious game called The Second Kind of Life with HIV (SKLWH) [9] was created for the HIV/AIDS education of students. Through role-playing in simulated life scenarios, the game allows players to experience the discrimination faced by people living with HIV. Quantitative and qualitative methods, that is, a randomized controlled trial and focus groups, respectively, were applied to evaluate the educational game. This research proposes the participatory serious game development model (PSGDM) and integrates game development and effect evaluation. By doing so, it aims to make up for the current paucity of studies on independent games, health function games, and other serious games in Chinese academia [10,11]. In particular, this research wishes to address the lack of effect evaluations of HIV/AIDS educational games based on randomized controlled trials [12].

### HIV-Related Stigma

Stigma refers to labeled differences. It is the result of social construction. As Link and Phelan have noted, everyone is different, but when certain differences are associated with negative ideas, they separate “us” from “them,” resulting in the loss of social status and discrimination for the individuals who are negatively labeled [13]. Stigma is widespread in social life. It is an ancient dilemma for humanity, involving race, ethnicity, gender, religion, health, and many other factors. HIV-related stigma belongs to the realm of health. Its wide prevalence and social consequences are considered “the third phase of the HIV pandemic” [14].

HIV-related stigma is one of the major obstacles to the prevention and control of HIV/AIDS. This problem is usually discussed from different sides to reflect the discrimination that people living with HIV encounter in various aspects of life, such as society’s moral condemnation of people living with HIV [15,16], the stigma they experience in their personal interactions [15-17], and the prejudices against intimate relationships with them [18-21]. Accordingly, this research discusses HIV-related stigma at the level of morality, personal interaction, and intimacy. Based on the logic of the social distance scale [22], marriage intention best reflects people’s acceptance of a certain group, while marriage resistance is the hardest to overcome. Moreover, empirical studies have shown that among the items that measure HIV-related stigma, the most frequently chosen one is refusing to have a date with a person living with HIV [20]. Therefore, we formulated the following 2 research hypotheses: (1) HIV-related intimacy stigma is greater than HIV-related morality stigma (hypothesis 1); and (2) HIV-related intimacy stigma is greater than HIV-related personal interaction stigma (hypothesis 2).

### Effects of Serious Games on HIV-Related Stigma

Sontag has argued that AIDS metaphors that generate fear and inflict stigma must be exposed, criticized, belabored, and used up [23], and this cannot be done without media communication. Since the early 1990s, the mass media have engaged in HIV/AIDS prevention and control, and they are now regarded as an effective “vaccine” against HIV infection. Among various media forms, serious games advocate the idea of entertainment education, expose social injustice, and reflect on discrimination; they are thus an effective intervention for communicating prosocial information [24]. HIV/AIDS educational games can be classified into many types, including racing games (eg, Fast Car: Travelling Safely Around the World), board games (eg, Make a Positive Start Today!), card games (eg, Mieux connaître les IST/VIH/SIDA), hero combat games (eg, AIDS Fighter · Health Defense), and narrative games (eg, Tumaini). SKLWH, the trial material used in this research, is an interactive narrative game.

Based on the entertainment overcoming resistance model (EORM), entertainment education media can overcome resistance from the audience by way of narratives, thus having a more positive effect on persuasion compared to traditional educational methods [25,26]. Digital games enable learning from interactive experience, which no other interventions can match [27]; they can also change attitude and behavior in an engaging environment [24]. The entertainment education strategy of serious games can make HIV/AIDS-related experiences more perceptible and familiar to players. They can thus mitigate discrimination through players’ identification with people living with HIV. Overall, it is predicted that games have a more significant effect on decreasing HIV-related stigma. The following 3 hypotheses were thus proposed: (1) After the intervention, the game groups are more tolerant than the control groups in terms of morality stigma (hypothesis 3); (2) After the intervention, the game groups are more tolerant than the control groups in terms of personal interaction stigma (hypothesis 4); and (3) After the intervention, the game groups are more tolerant than the control groups in terms of intimacy stigma (hypothesis 5).

A great number of issues related to sex exhibit gender differences. HIV/AIDS interventions do not have the same effect on different genders [28]. Existing studies indicate that females show less discrimination against people living with HIV than males [18,29,30]. However, one study suggested that females are more tolerant of people living with HIV than males in terms of morality stigma and personal interaction stigma, but that there is no significant gender difference regarding intimate interaction stigma [21]. Given this evidence, we formulated the following 2 hypotheses: (1) Females are more tolerant of people living with HIV than males in terms of morality stigma (hypothesis 6); and (2) Females are more tolerant of people living with HIV than males in terms of personal interaction stigma (hypothesis 7).

## Methods

### Method 1: Game Development

Based on the EORM, SKLWH was designed between August 2018 and April 2019 by 5 master’s students using free resources of Cheng Guang, a popular user-generated content game website in China, under the supervision of the author XZ. The game is freely accessible from computers and smartphones. Digital technology means for HIV/AIDS education represent both an intervention delivery tool and a research tool [31]. Through gamification, social experiments can be conducted [32]. From this perspective, games can be seen as an “alternative laboratory” that helps break limited mindsets [33]. As a research method, serious gamification aims to convert the idea of entertainment education into practice, which involves constructing external and internal game grammar. According to Sun [34], external game grammar is related to specific social practices and identities, while internal game grammar is related to narrative elements in the form of games. The game development of this study involved designing and combining the external and internal grammar of SKLWH, and then, the game’s effect was evaluated with a randomized controlled trial and focus group discussions.

### Method 2: Experimental Evaluation

#### Research Procedure

The researchers invited university students to fill in a recruitment questionnaire through WeChat, China’s largest social media platform. A total of 167 students from 7 universities in Guangzhou were recruited to voluntarily participate in the offline experiment from May 25 to June 2, 2019, and on March 21, 2021. The participants were aged between 17 and 28 years, with an average age of 20 years. They were allocated to the game and control groups using random numbers. A total of 27 participants were excluded during the data cleaning process (25 had already accessed the material and 2 provided careless responses). Data from the remaining 140 participants were obtained and deemed effective, as the sample size was larger than the minimal requirement of 128 (α=.05, power=0.8, effect size=0.25), which was calculated with G*Power (University of Dusseldorf). Finally, the trial had the following 4 groups: female game group (36 participants), male game group (36 participants), female control group (34 participants), and male control group (34 participants).

The game SKLWH served as the media intervention for the treatment groups. Its main content was converted into pictures and text, and sent to the control groups through WeChat posts. Both the game groups and control groups used smartphones to access their materials, thus ensuring consistency of presentation. The students did not know what kind of materials they would be exposed to before the trial. Each participant either played the game or read the WeChat posts, and immediately afterward, they filled out a questionnaire. There was no trial registration for this study, because when the authors started recruitment in April 2019, their university did not have such a requirement or tradition for social science disciplines. Moreover, when the authors started recruitment, their university did not have an ethical committee for the social sciences. As with a previous study [35], ethical rules were observed as much as possible in the course of this research. Each subject participated after providing informed consent, and was informed of privacy protection and the right to withdraw from the trial at any time.

#### Measures

The dependent variable of this research was HIV-related stigma, which involves 3 dimensions: morality stigma, personal interaction stigma, and intimacy stigma. The short HIV stigma scale was used to assess HIV-related stigma. This scale has 12 items, and each item uses a 4-point Likert scale (1, “strongly disagree” to 4, “strongly agree”). Some items were recoded, with higher scores indicating higher degrees of stigma. Missing values were replaced with means. This research reduced the scale’s dimensionality via principal component analysis. The Kaiser-Meyer-Olkin value of the factor analysis was 0.82, and the Bartlett test of sphericity was significant (*P*<.001). Three dimensions were identified after deleting 2 items, which cumulatively explained 72.68% of the variance. The first dimension was personal interaction stigma (Cronbach α=.86), and a sample statement for this was “People living with HIV should be allowed to study with others.” The second dimension was intimacy stigma (Cronbach α=.90), and a sample statement for this was “I’m not willing to marry a person living with HIV.” The third dimension was morality stigma (Cronbach α=.64), and a sample statement for this was “Only those infected by HIV via blood transfusions or injections in hospitals deserve care and treatment.”

### Method 3: Focus Group Discussions

Immediately after the experimental evaluation, the researchers conducted group discussions to obtain feedback on SKLWH and HIV-related stigma. Sixty-four trial participants were invited to form 16 groups for discussion (Table 1) before the data reached saturation and new information could not be found. Participants in groups 1 to 9 played the game, while those in groups 10 to 16 read the WeChat posts. With the consent of the participants, the discussions were recorded and transcribed for further analysis.

**Table 1:** Composition of the focus groups.

Group	Number of participants	Gender of participants
Group 1	3	One male and two females
Group 2	5	Five females
Group 3	2	One male and one female
Group 4	6	One male and five females
Group 5	2	One male and one female
Group 6	5	Two males and three females
Group 7	3	Three males
Group 8	2	Two males
Group 9	2	Two males
Group 10	5	Two males and three females
Group 11	6	Three males and three females
Group 12	6	Two males and four females
Group 13	2	One male and one female
Group 14	6	Four males and two females
Group 15	5	One male and four females
Group 16	4	Four males

The discussion centered on the following questions: Which is your favorite channel to receive HIV/AIDS education—games, WeChat, or lectures? Why do you prefer this channel? In your opinion, what kinds of situations are people living with HIV facing in society?

Participants in groups 1 to 9 who played SKLWH were also asked the following questions: Is SKLWH attractive to you? If so, why? In your opinion, is SKLWH effective for HIV/AIDS education? Did you identify with Qin Qin, the protagonist of SKLWH, when playing the game? How can SKLWH be improved?

## Results

### Game Development Results

#### External Grammar of SKLWH

**Figure 1:**
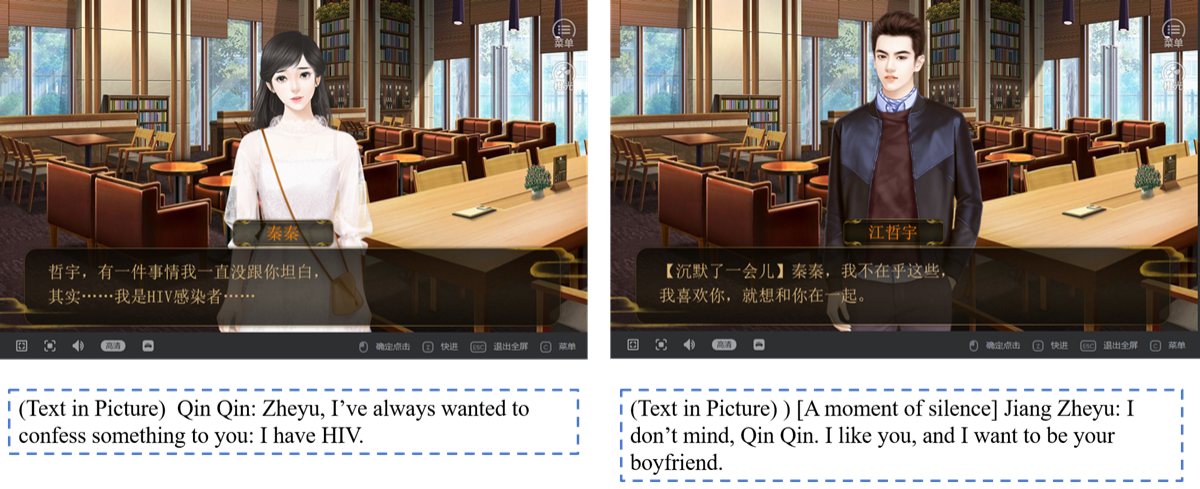
A character not living with HIV expresses his feelings toward a character living with HIV (protagonist).

The experience of serious game playing is a process of value-based learning [36]. As SKLWH is a serious game reflecting on HIV-related stigma, the values constructed in its text are of great importance. After collecting extensive original stories and consulting experts in HIV/AIDS education, the design team decided that the game would reflect the stigma faced by people living with HIV from the perspective of morality, personal interaction, and intimacy, with an emphasis on the idea that people’s right to marry and have romantic relationships should not be hampered by disease. The main character of the game is Qin Qin, a female college student who has HIV. By taking the role of Qin Qin, players can experience the life of people living with HIV. In SKLWH, a character not living with HIV called Jiang Zheyu likes Qin Qin and confesses his affection for her. Qin Qin then reveals her HIV status, but Jiang Zheyu does not give up his feelings for her (Figure 1). Qin Qin (ie, the player) finally decides whether to marry Jiang Zheyu. As an expert said, this kind of story happens in real life and is in accordance with China’s legal guidelines [37]. However, despite improvements in legal and medical protection, it is still difficult to mitigate the loss of life chances in general and intimacy rights in particular of people living with HIV.

#### Internal Grammar of SKLWH

**Figure 2:**
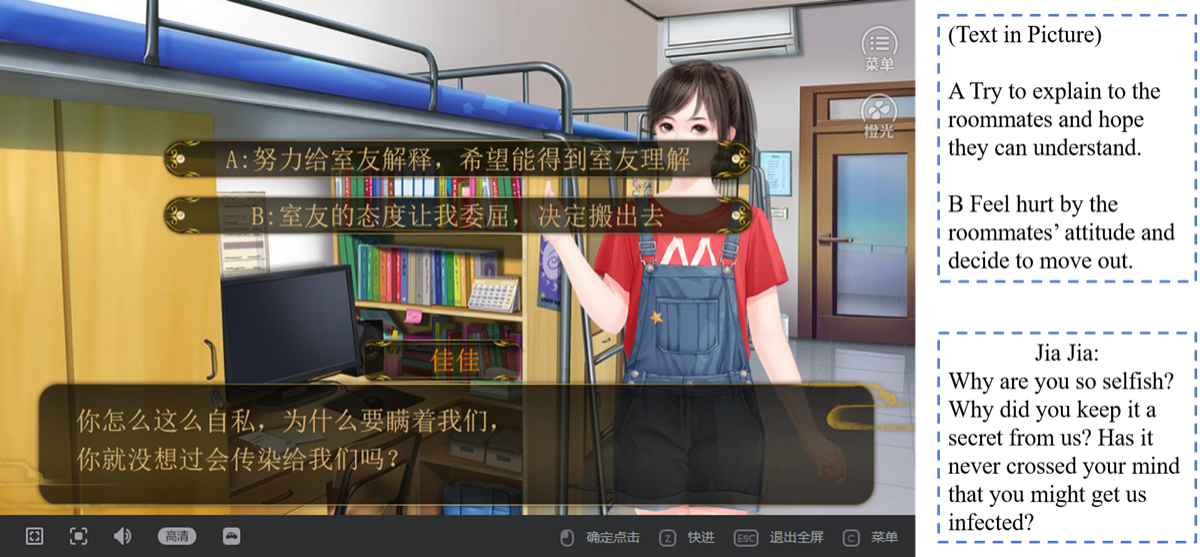
An instance of interactive decision-making in the game.

The external game grammar of SKLWH is built on its internal game grammar, including narrative elements and game materials. The narrative elements involve the following 4 aspects:

1. Game-character setting: The principle of setting characters is telling a complete story with only a few characters to make sure that the persuasion effect is not jeopardized by a lengthy game.
2. Game-structure layout: The classic structure of serious games is “trunk plots-branch plots-trunk plots,” meaning that the game starts from the same (trunk) plot, enters different branch plots based on different decisions, and goes back to the trunk plot after the branch plots come to an end.
3. Decision-making setting: A decision-making plot is one where players enter different branch plots depending on their choices. Some decision-making plots are set to reflect the themes of serious games, while others are set to enhance playfulness. Figure 2 shows a decision-making plot where the protagonist must choose whether to move out of the dormitory when her HIV status is discovered by her roommates.
4. Ending setting: In interactive narrative games, players encounter many moments of decision-making, and different combinations of decisions lead to different plots and endings. In SKLWH, players’ decisions eventually lead to 6 different endings, which condense the reflection on and breaking of HIV-related stigma.

**Figure 3:**
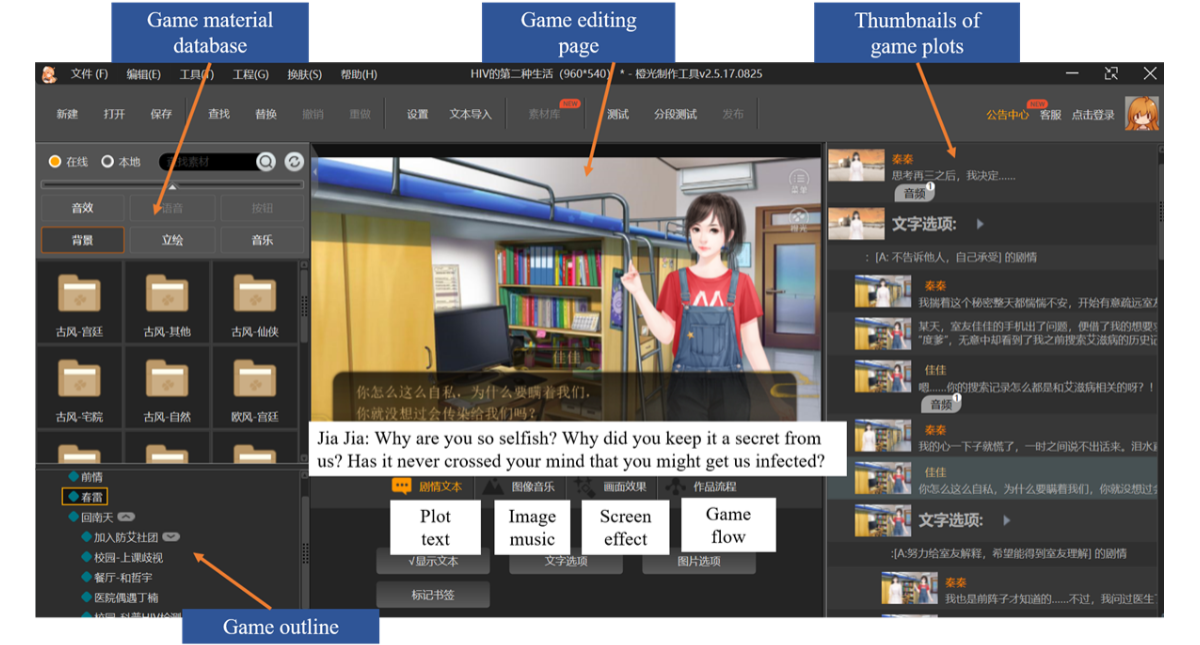
Screenshot of the game in the process of being developed.

**Figure 4:**
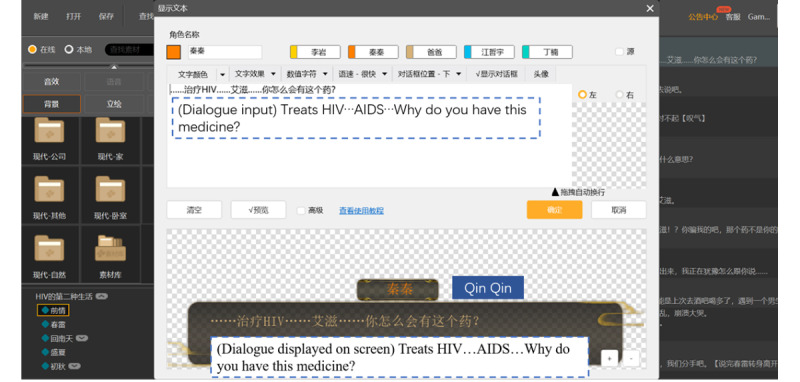
Dialogue input of the game in the process of being developed.

Using the free materials in Cheng Guang’s database, game developers without professional training can easily actualize game narratives by creating scenarios, dialogues, decision-making settings, special effects, and so on. Figure 3 shows the development process of the above interactive decision-making plot. Figure 4 illustrates how to input a dialogue during the game development process.

#### Participatory Serious Game Development Model

The section above briefly outlined the development process of SKLWH. Young students’ active engagement in game development reflects the idea of “participatory culture” advocated by the communication scholar Henry Jenkins [38]. This idea entails that amateur developers participate as citizens, use technical platforms with low barriers to entry, and assume the responsibility of preventing and controlling HIV/AIDS through gamification.

**Figure 5:**
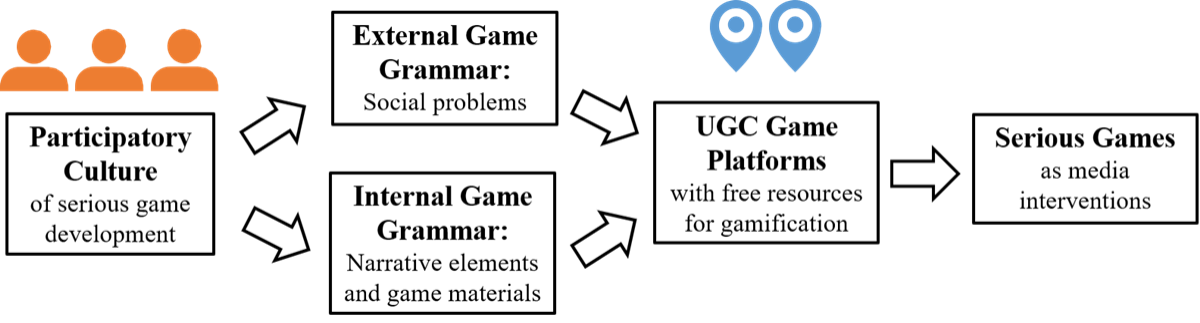
The participatory serious game development model. UGC: user-generated content.

Among the few HIV/AIDS education games in China, SKLWH is special because its development model can be popularized. It is a good example of the PSGDM proposed in this article (Figure 5). In this model, developers set the external game grammar by capturing important social issues and set the internal game grammar by means of narrative elements and game materials. The 2 grammars are combined in user-generated content game platforms, such as China’s Cheng Guang, Yi Ci Yuan, and Tencent Games, as well as the overseas platform Steam. This means that the PSGDM can be used both inside China and abroad, allowing extensive community participation in game development and easy access to the public wishing to play the developed games.

**Figure 6:**
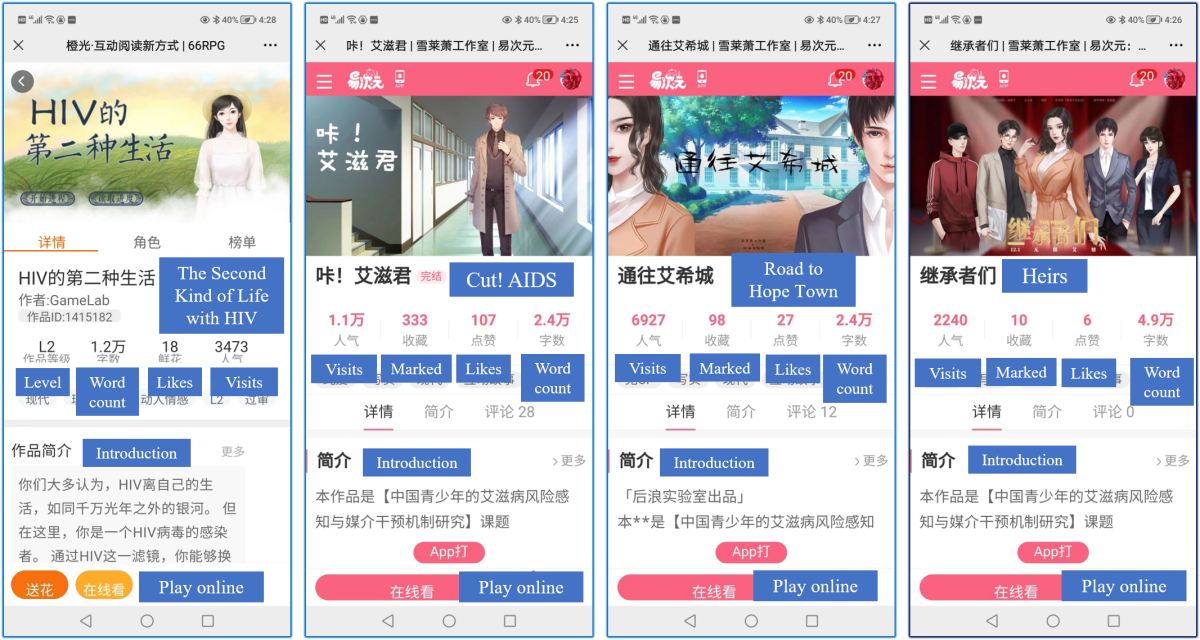
Mobile interfaces of HIV educational games developed based on the participatory serious game development model.

Besides SKLWH, the team of the author XZ developed 3 other HIV-related games following the PSGDM and launched them on Yi Ci Yuan between 2020 and 2022, namely Cut! AIDS, Road to Hope Town, and Heirs (Figure 6). SKLWH is a love story, the protagonist of which is a female college student who has HIV. Cut! AIDS is a film-production story in which 3 male protagonists are infected with HIV or are at risk of HIV infection. These 2 games mainly reflect the HIV-related stigma against different groups. By telling a detective story, Road to Hope Town reflects not only external stigma toward people living with HIV but also the internalized stigma of people living with HIV. Reflecting HIV-related stigma as well as AIDS phobia, Heirs renarrates Road to Hope Town’s detective story and allows players to identify with either a protagonist living with HIV or a protagonist not living with HIV. Regarding the external grammar, these 4 games work together to comprehensively reflect the complex dynamics of HIV stigma. Concerning the internal grammar, the playfulness of the love, film production, and detective stories was strengthened via interactive mechanisms, including decision-making, questions and answers, point rewards and punishments, clue search, and task accomplishment.

### Experimental Evaluation Results

#### Degree of HIV-Related Stigma

First, the degree of HIV-related stigma in different dimensions was compared to explore if intimacy stigma is more evident than the other dimensions of stigma. Paired sample *t* tests (Table 2) showed that intimacy stigma (mean 2.58, SD 0.83) was far greater than morality stigma (mean 1.40, SD 0.46), with a significant difference between the 2 means (t_139_=15.0; *P*<.001). Intimacy stigma was also greater than personal interaction stigma (mean 1.36, SD 0.55), with a significant difference between the 2 means (t_139_=16.97; *P*<.001). However, there was no significant difference between the mean values of morality stigma and personal interaction stigma (t_139_=0.76; *P*=.45).

**Table 2:** Paired sample *t* tests of HIV-related stigma (N=140).

Variable	*t* value (*df*)	Difference between means	*P* value
Intimacy stigma- morality stigma	15.0 (139)	1.18	<.001
Intimacy stigma- personal interaction stigma	16.97 (139)	1.22	<.001
Morality stigma- personal interaction stigma	0.76 (139)	0.04	.45

#### Effects of Media Interventions

As HIV/AIDS interventions may not have the same effect on different genders [28], the impact of media interventions and gender on different dimensions of HIV-related stigma were explored by 2-way analysis of covariance. Chinese students usually get information about HIV/AIDS education from lectures and WeChat. Games are not a common channel, but once applied, they leave a deep impression. Therefore, the exposure frequencies of the 3 interventions were controlled as covariates. In terms of morality stigma, neither the interaction effect (*F*_1,133_=0.05; *P*=.82) nor the main effect of media interventions (*F*_1,133_=2.52; *P*=.11) was significant. The main effect of gender was significant (*F*_1,133_=6.60; *P*=.01), with females’ discrimination against people living with HIV (mean 1.29, SD 0.39) being lower than that of males (mean 1.50, SD 0.51). In terms of personal interaction stigma, the interaction effect (*F*_1,133_=0.89; *P*=.35) was not significant. Furthermore, neither the main effect of media interventions (*F*_1,133_=0.04; *P*=.85) nor that of gender (*F*_1,133_=2.17; *P*=.14) was significant. Regarding intimacy stigma (Table 3), neither the interaction effect (*F*_1,133_=0.38; *P*=.54) nor the main effect of gender (*F*_1,133_=0.55; *P*=.46) was significant. However, the main effect of media interventions was significant (*F*_1,133_=4.31; *P*=.04), and the effect of decreasing intimacy stigma was stronger in the game groups (mean 2.43, SD 0.84) than in the control groups (mean 2.73, SD 0.80). Thus, hypotheses 1, 2, 5, and 6 were verified. Intimacy stigma was the most severe among the 3 dimensions of HIV stigma. Females were more tolerant than males with regard to morality stigma, and the game intervention had an advantage in decreasing intimacy stigma. In all the analyses, the 3 covariates were not significant. The interactions were also not significant, and the effect of media interventions on all the dimensions of HIV-related stigma did not vary according to gender.

**Table 3:** Two-way analysis of covariance of intimacy stigma (N=140).

Variable	*F* value (*df*)^a^	*P* value
Pretrial lecture exposure	0.39 (1,133)	.53
Pretrial WeChat exposure	0.04 (1,133)	.84
Pretrial game exposure	0.41 (1,133)	.52
Media intervention	4.31 (1,133)	.04
Gender	0.55 (1,133)	.46
Media intervention × gender	0.38 (1,133)	.54

^a^Corrected total=139.

### Group Discussion Results

#### Discussion Themes

The discussion themes were analyzed using NVivo (QSR International), which is a popular software for organizing, analyzing, and managing qualitative data [39], to explore the students’ patterns of understanding (Table 4).

**Table 4:** Focus group themes.

Theme and subtheme				Reference frequency, n (%)
**Favorite education channel (N=56)**				
	Games (13 groups)			33 (58.9)
	WeChat (10 groups)			18 (32.1)
	Lectures (5 groups)			5 (8.9)
**Defects of SKLWH^a^ (N=56)**				
	Unattractiveness (5 groups)			9 (16.1)
	Plot (6 groups)			22 (39.3)
	HIV-related information (6 groups)			8 (14.3)
	Interactivity setting (7 groups)			17 (30.4)
**Advantages of SKLWH (N=51)**				
	Attractiveness (8 groups)			18 (35.3)
	Game design (4 groups)			8 (15.7)
	Educational effect (7 groups)			22 (43.1)
	Innovation (3 groups)			3 (5.9)
**Identification with the protagonist (N=42)**				
	Yes (9 groups)			28 (66.7)
	No (7 groups)			14 (33.3)
**HIV-related stigma (N=102)**				
	**Morality stigma (12 groups)**			28 (27.5)
		Indiscreet behavior (9 groups)	15 (14.7)	
		Malicious transmission (8 groups)	13 (12.8)	
	Personal interaction stigma (10 groups)			23 (22.5)
	Intimacy stigma (11 groups)			25 (24.5)
	General statement (9 groups)			26 (25.5)
Internalized stigma (3 groups) (N=7)				7 (100)
Fear of people living with HIV (3 groups) (N=3)				3 (100)

^a^SKLWH: The Second Kind of Life with HIV.

#### Participant Narratives

Thematic coding indicated that the game intervention was more attractive to students. Among the 56 participants who answered the question regarding the favorite channel to receive HIV/AIDS education, 33 (59%) chose games, 18 (32%) chose WeChat, and 5 (9%) chose lectures. In the extracts below, 2 male participants from groups 7 and 13 explained their reasons for preferring games:

Personally, I prefer to choose games. [Games are] more interesting. Lectures can be very boring, and [I am usually] lazy in opening WeChat posts due to their low readability.

Game intervention participant #29

In my opinion, the most interesting form is games, because there is a sense of participation. If I have to rank [the channels], I will say games, WeChat posts, and lectures.

WeChat intervention participant #18

For some participants, SKLWH was unattractive. In their opinion, it required an improved plot, more useful HIV-related information, and better interactivity settings. However, these participants compared SKLWH to commercial games from the entertainment industry rather than to other HIV educational tools. Those participants who liked the game praised its plot and educational effects. SKLWH was also sometimes praised for its innovation in the field of HIV/AIDS education. A female respondent in group 3 made the following comment:

This game largely appealed to me. In contrast, the lectures on HIV/AIDS education that I heard before were very boring. The game is useful to disseminate HIV-related information through storytelling.

Game intervention participant #11

“What kinds of situations are people living with HIV facing in society?” was a broad question that allowed participants to answer according to their understanding. Many participants discussed various kinds of stigma faced by people living with HIV. They also described the behavior of people living with HIV as indiscreet and criticized “some” people living with HIV for maliciously spreading HIV on purpose. Ignored by previous studies, “malicious transmission” is also an indicator of morality stigma. This can be shown through the following dialogue between 2 female participants in group 1:

There are some [people living with HIV], especially those who are anti-social

persons]…[Game intervention participant #1

They do not confess [their HIV-positive status] and intentionally have unprotected sex to disseminate HIV.

Game intervention participant #3

That’s right.

Game intervention participant #1

As mentioned earlier, the paired sample *t* test showed that intimacy stigma was significantly greater than morality stigma and personal interaction stigma. Indeed, the impossibility of intimacy was “a matter of principle” for some participants, as shown by the following explanation offered by a male student in group 3:

People living with HIV are discriminated at the social level. As far as I am concerned, I do not discriminate against people living with HIV and can accept them, but [I’m a person] with principles and a baseline.

Game intervention participant #10

Could you please explain what constitutes a matter of principle for you?

Researcher

For example, I don’t discriminate against people living with HIV and can dine with them, but it is unacceptable to have a romantic relationship [with them].

Game intervention participant #10

This is a principle for you.

Researcher

Some people may think I discriminate against people living with HIV.

Game intervention participant #10

[The intimacy issue] was raised in the questionnaire.

Researcher

Yes, I cannot cross that line, that is, I cannot accept [having romantic relationships] with people living with HIV. But I am not a person who discriminates against people living with HIV.

Game intervention participant #10

The dialogue below between 1 female and 2 male students in group 10 reflects morality stigma, personal interaction stigma, and intimacy stigma simultaneously.

I think it does not matter for me to have everyday interactions or communications with people living with HIV, as long as [intimate relationships] are not concerned.

WeChat intervention participant #5

As far as I am concerned, I can have personal contacts [with people living with HIV]. But if my relatives and friends knew it, they would say, “Oh, how dare you stay with those people living with HIV? Don’t you worry about being infected?” Then, I may not dare to have interactions [with people living with HIV].

WeChat intervention participant #4

As regards romantic relationships with people living with HIV and so on, as listed in the questionnaire, I am quite unwilling to have them. But it is okay for me to have common interactions. Moreover, in the opinion of most people, HIV infection means that people living with HIV are indiscreet in their private life.

WeChat intervention participant #2

Regarding identification, 67% (28/42) of the related narratives were about the identification experience with the character living with HIV, while 33% (14/42) were not. A female student in group 4 described her feelings when playing the game:

I had a sense of identification. I hesitated a little when making choices. I imagined what I would choose if I were the female protagonist.

Game intervention participant #18

It is worth noting that some participants reported complicated game experiences, as they sometimes identified with Qin Qin and sometimes did not. Game intervention participant #12, another female student in group 4, regarded herself as unidentified. However, she followed the plot and carefully considered the interactive decisions. Therefore, identification is an important construct and should be explored in future studies of the stigma-decreasing effect of game interventions.

## Discussion

### Principal Findings

In the past, when intervention mechanisms, such as serious games, were expected to reduce HIV-related stigma, the stigma was usually viewed as a whole. It was unclear which dimensions of HIV stigma could be alleviated by games or other factors. By closely examining 3 dimensions of HIV stigma, this research found that females have a more tolerant attitude than males in the dimension of morality stigma. With regard to decreasing personal interaction stigma, no significant difference was found between the 2 genders or the 2 media interventions. The game intervention had a greater effect in reducing intimacy stigma, which was strong and difficult to mitigate, with both genders’ attitudes remaining conservative regarding this issue. The nonsignificant interaction effect demonstrates that the effect of media interventions does not vary with gender. Accordingly, our focus should not be on designing different versions of educational content for each gender but on decreasing different dimensions of HIV-related stigma with the most effective media intervention, such as using games to decrease intimacy stigma.

By providing in-depth information, the findings of the group discussions validated the results of the quantitative analysis. Intimacy stigma was much more severe than morality stigma or personal interaction stigma. The impossibility of intimacy is a principle that was often expressed by participants when considering their relationships with people living with HIV. The game intervention was largely praised in terms of HIV/AIDS education, though SKLWH did not satisfy all participants. According to some of them, one of the main defects of the game was the optimistic description of the life of a person living with HIV, especially the romantic relationship between the protagonist living with HIV and the character not living with HIV. In their view, this kind of life is abnormal and unrealistic. In fact, this criticism reflects the deep discrimination against people living with HIV. Instead, SKLWH hopes to publicize that people living with HIV can live a normal life, that is, a second life different from that imagined by the public.

Furthermore, to advance research beyond individual cases, this study provides the following insights into the development of serious games. First, this research is an example of the development and effect evaluation of HIV/AIDS educational games in China, which can encourage more relevant practices in the future. Second, the PSGDM proposed in this study can promote a participatory culture for developing serious games by those dedicated to health communication and media interventions. Third, as an interdisciplinary study on communication and medicine, this research shows that HIV/AIDS prevention or control is not the duty of only medical experts and that serious game development is not the responsibility solely of game experts. More people can be engaged in and share the responsibility of health education.

### Strengths and Limitations

This study linked game development and effect evaluation; it used a mixed methods evaluation to achieve data triangulation and combined causality verification and in-depth explanation. SKLWH and 3 other games developed by the team of the first author XZ help fill the gap in web-based gaming tools for HIV/AIDS education in China. With around 26,000 visits to date, the 4 games have tried to extend their educational effect among young people. Although the games are far from perfect, the gamification practices based on the PSGDM are very promising.

The team was not very experienced when they developed SKLWH, and the game is thus relatively simple in form and not sufficiently entertaining. As a result, the desired persuasive effect on attitudes based on the EORM has not been fully realized. The measurement of HIV-related stigma also needs improvement, and the reliability of morality stigma in this study was not high enough. The HIV-related stigma scale should be modified in light of current social mentalities and concrete contexts. Future studies may consider including the malicious consciousness of HIV transmission of people living with HIV as an indicator of morality stigma, as suggested in the group discussions. Moreover, controlling the theoretical variables could lead to a better analysis of the mechanisms in the game’s effect.

### Comparison With Prior Work

One of the few effect evaluations of HIV/AIDS educational games in China found that the hero combat game AIDS Fighter · Health Defense did not have a significant effect on decreasing HIV-related stigma [40]. The researchers suggested that the decrease in stigma may need time to take effect. In addition, we should also notice the differences among various types of serious games. Racing games, board games, card games, hero combat games, and narrative games are all applicable to HIV/AIDS education. However, narrative games may have a greater effect on mitigating stigma owing to their narratives and mechanisms, such as empathy, identification, and transportation [24].

### Conclusions

Among the different methods of HIV/AIDS education that target young students, games are a feasible and highly acceptable choice. Due to the lack of a cure and an effective vaccine for AIDS, this disease needs games and other forms of media interventions more than other infectious diseases. More effort should be made in serious game development for HIV/AIDS education, and SKLWH is one of the few results of this effort in China. By leveraging free resources on digital platforms, new media users can develop games for health education by themselves, thereby enabling extensive social engagement in HIV/AIDS prevention and control. People living with HIV experience being regarded as “others.” Society rejects them, and sometimes they even reject themselves. If this problem remains unsolved, it could cause difficulties in the treatment, prevention, and control of HIV/AIDS. The suffering of “others” leads to the regression of society. We need more serious games to allow players to empathize with people living with HIV and reflect on HIV-related stigma. Only this can allow us to completely break the metaphor of “others.”

## Abbreviations

EORM: entertainment overcoming resistance model
PSGDM: participatory serious game development model
SKLWH: The Second Kind of Life with HIV
